# Glucose-derived AGEs enhance human gastric cancer metastasis through RAGE/ERK/Sp1/MMP2 cascade

**DOI:** 10.18632/oncotarget.22185

**Published:** 2017-10-31

**Authors:** Ruyuan Deng, Fengbo Mo, Bowen Chang, Qi Zhang, Hui Ran, Shuhua Yang, Zhiqiang Zhu, Lei Hu, Qing Su

**Affiliations:** ^1^ Department of Endocrinology, Xinhua Hospital, Shanghai Jiaotong University School of Medicine, Shanghai, China; ^2^ Department of Orthopaedic Surgery, Union Hospital, Tongji Medical College, Huazhong University of Science and Technology, Wuhan, China; ^3^ Department of Surgery and Orthopaedic Surgery, Massachusetts General Hospital and Harvard Medical School, Boston, MA, USA; ^4^ Department of General Surgery, Renji Hospital, Shanghai Jiaotong University School of Medicine, Shanghai, China; ^5^ Department of General Surgery, Anhui Provincial Hospital of Anhui Medical University, Hefei, China; ^6^ Shanghai Key Laboratory of Gastric Neoplasms, Shanghai Institute of Digestive Surgery, Ruijin Hospital, Shanghai Jiaotong University School of Medicine, Shanghai, China

**Keywords:** AGEs, RAGE, Sp1, MMP2, metastasis

## Abstract

Advanced glycation end products (AGEs) have been reported to take part in many cancer processes. Whether AGEs contribute to gastric cancer (GC) course and the underlying mechanism are still unclear. Here, glucose-derived AGEs are detected to be accumulated in tumor tissues and blood of patients with GC. As the receptor for AGEs, RAGE is highly expressed in cancer tissues, and closely associated with the depth of cancer invasion, lymph node metastasis and TNM stage. Both *in vivo* and *in vitro* treatment of AGEs accelerate the tumor invasion and metastasis, with upregualtion of RAGE, Specificity Protein 1 (Sp1), and MMP2 protein expression, as well as enhancement of MMP2 activity. Either RAGE-blocking antibody or Sp1-knockdown can partially block the AGEs-induced effects. Moreover, AGEs increased the phosphorylation of ERK, and reducing the phosphorylation level of ERK by MEK1/2 inhibitor decreased the expression of Sp1. These results indicate that accumulation of glucose-derived AGEs may act as one of potential risk factors for GC progression and promote the invasion and metastasis of gastric cancer partially through the activation of RAGE/ERK/Sp1/MMP2 pathway.

## INTRODUCTION

Both morbidity and mortality of gastric cancer (GC) are very high among all the cancers worldwide. Many pathological factors are correlated with the development and advance of GC, such as Helicobacter pylori infection, smoking, and inflammation [1]. The identifications of unknown etiological factors and underlying mechanism are crucial for prevention and treatment of GC.

As products of a non-enzymatic reaction, where lysine residues are glycation, advance glycation end products (AGEs) are implicated in progressions of age-related diseases, diabetic complications, as well as cancer course [2-4]. Crosslinking with matrix proteins and binding to the cell surface receptors, especially the receptor for AGEs (RAGE), are the main ways for AGEs to exert their pathological and physiological effects [5, 6]. The combination of AGEs and RAGE can activate different signaling pathways including NF-κв, MAPK, or Jak/Stat pathways in different tissues or cells to be implicated in inflammation, proliferation, and motility [7, 8]. RAGE signaling has been reported to be involved in invasion and metastasis of GC [9]. However, the underlying mechanism of AGEs-RAGE interaction-induced GC metastasis remains to be further studied.

As the member of Sp family, Specificity Protein 1 (Sp1) is overexpressed in a variety of cancers, such as gastric cancer, pancreatic cancer, and prostate cancer, and is closely associated with poor prognoses of cancers. By regulating the expression of effector molecules including SREBP-1c, VEGF, MMP2, Sp1 plays pivotal roles in cancer cell metabolism, proliferation, and metastasis [10]. However, factors which regulate Sp1 expression in cancers and the underlying mechanism are still unclear. Interestingly, silencing of RAGE in colorectal cancer cells resulted in decreasing of Sp1 expression, which suggested that RAGE signaling may regulate Sp1 expression.

Here, we design a series of experiments to investigate the role of glucose-derived AGEs on the invasion and migration of gastric cancer. AGEs were found to be accumulated in blood of GC patients and gastric cancer tissues. AGEs-RAGE signaling cascade induced the cancer cell invasion and migration partially through increasing expressions of RAGE, Sp1, and MMP2. Our results uncover glucose-derived AGEs as a new risk factor for gastric cancer, which may help us improve the progression and prognosis of gastric cancer.

## RESULTS

### Accumulation of glucose-derived AGEs in sera and cancerous tissues and high expression of RAGE, Sp1, and MMP2 in tumor in gastric cancer patients

**Figure 1:**
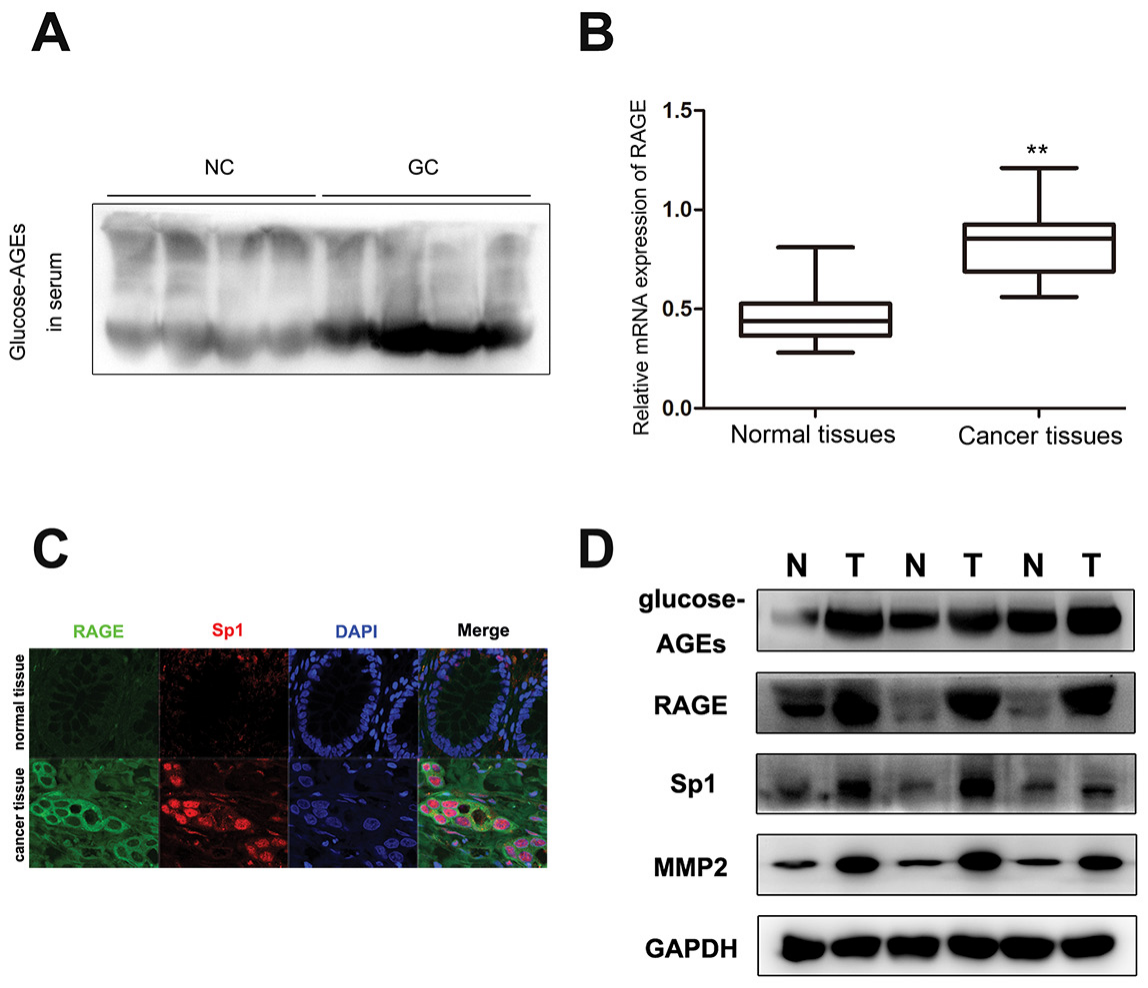
The content of glucose-derived AGEs in sera and cancer tissues, as well as coexpression of RAGE, Sp1, and MMP2 in gastric cancer tissues **(A)** The level of glucose-derived AGEs in serum determined by western blot. (NC: normal control, GC: gastric cancer patients). **(B)** The level of RAGE mRNA expression in gastric cancer tissues and paired normal tissues, normalized by GAPDH. **(C)** Co-expression of RAGE and Sp1 in cancer tissues detected by confocal. The scale bar is 25μm. **(D)** Accumulation of glucose-derived AGEs and overexpression of RAGE, Sp1, and MMP2 in tumor tissues assayed by western blot.

Commonly, AGEs are accumulated in diabetic patients [11]. Here, with age standardization, the serum concentrations of glucose-derived AGEs in GC patients without diabetes were found to be markedly increased compared with healthy people (Figure 1A). However, there was no significant difference in serum total protein between gastric cancer patients and normal control (Supplementary Figure 1). Meanwhile, as the receptor for AGEs, RAGE mRNA expression in cancer tissues was higher than in paired non-tumor tissues (Figure 1B). The result of tumor tissue immunofluorescence staining for 160 GC patients showed that RAGE expression was significantly higher in invasive cancer tissues than in non-invasive tumor tissues (Supplementary Figure 2). 98 out of 160 (61.25%) GC tissues showed RAGE positive staining. And the analysis of association between RAGE expression and clinicopathological factors of GC patients displayed that the increased expression of RAGE in GC tissues was positively related to lymph node metastasis (*p*=0.025) and TNM stage (*p*=0.004), but not to other factors (Table 1). The results of the multivariate regression analysis confirmed the increased expression of RAGE was significantly associated with lymph node metastasis and TNM stage. And as for age, RAGE expression was a significant independent prediction factor for metastasis in GC patients (Table 2). Furthermore, co-overexpression of RAGE, Sp1, and MMP2 (Figure 1C, 1D) and accumulation of AGEs in cancer tissues (Figure 1D) were found in invasive cancer tissues, which suggested that the accumulation of glucose-derived AGEs may contribute to gastric cancer progression by regulating the expression of RAGE, Sp1, and MMP2.

**Table 1:** Association between RAGE expression and clinicopathological factors of gastric cancer patients

Variables	Number of cases	RAGE immunofluorescence staining		*P*
		Positive (n=98)	Negative (n=62)	
Gender				
Male	107	64	43	0.596
Female	53	34	19	
Age (years)				
≥60	95	55	40	0.292
≥60	65	43	22	
Tumor differentiation				
Well to moderate	69	41	28	0.679
Poor	91	57	34	
Tumor location				
Gastric fundus	9	4	5	0.552
Gastric corpus	76	48	28	
Pylorus	75	46	29	
Tumor size				
≤3cm	75	43	32	0.339
≥3cm	85	55	30	
T stage				
T1+T2	46	22	24	0.027
T3+T4	114	76	38	
Lymph node metastasis				
Negative	70	36	34	0.025
Positive	90	62	28	
Distant metastasis				
Negative	151	91	60	0.295
Positive	9	7	2	
TNM stage				
I+II	53	24	29	0.004
III+IV	107	74	33	

Note: Positive RAGE expression included all positive cases, such as weak and strong.

**Table 2:** Multivariate regression analysis of RAGE expression and clinicopathological factors of gastric cancer patients

Variables	RAGE expression	
	*P*	HR (95% CI)
Age	0.053	0.372 (0.137-1.014)
Distant metastasis	0.552	0.443 (0.030-6.467)
T stage	0.951	1.033 (0.364-2.934)
Lymph node metastasis	0.000	0.090 (0.030-0.274)
TNM stage	0.001	0.180 (0.067-0.486)

### AGEs-RAGE enhance the gastric cancer peritoneal metastasis *in vivo*

**Figure 2:**
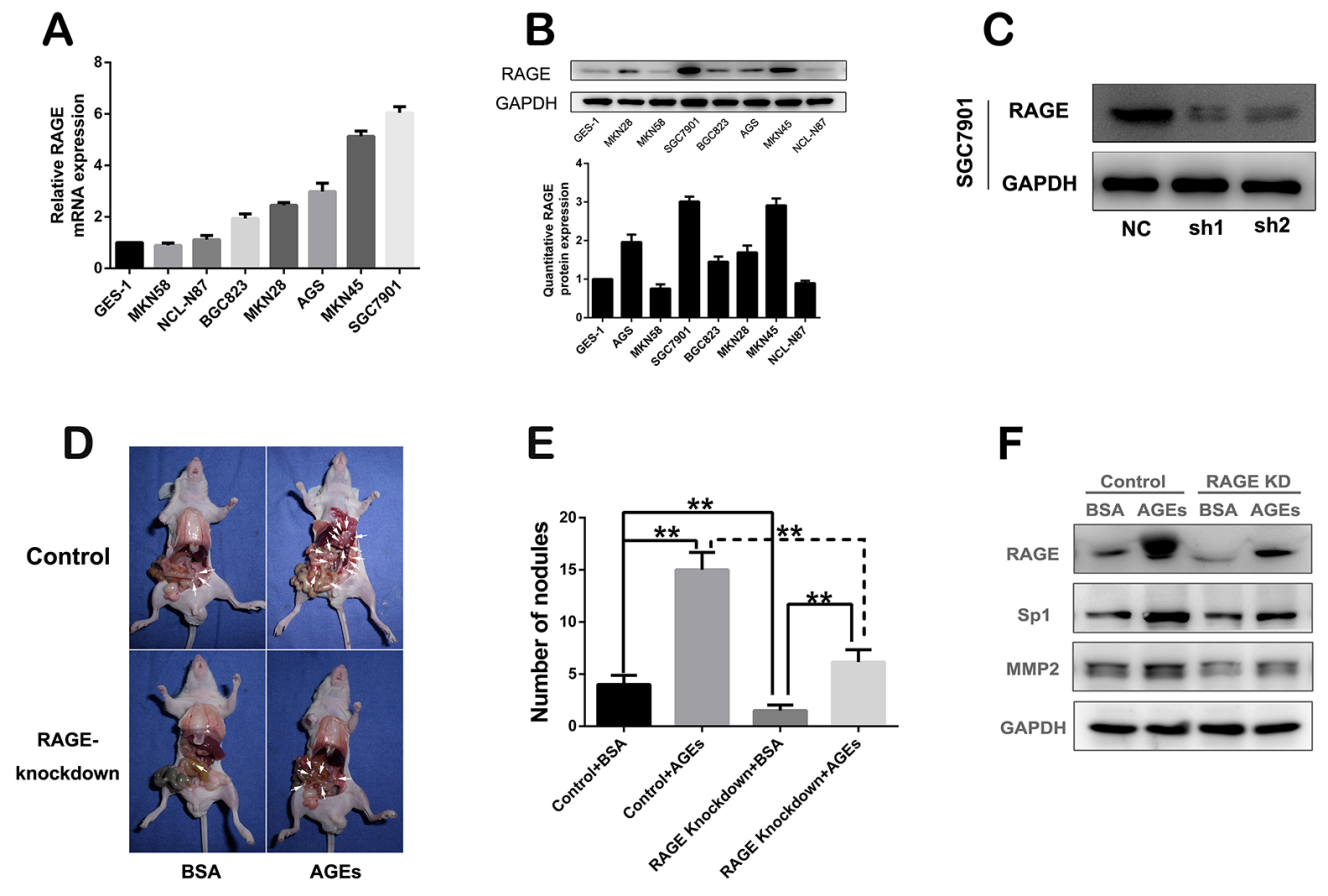
*In vivo* effect of glucose-derived AGEs on peritoneal spreading and metastasis of gastric cancer cells **(A)** mRNA expression profile of RAGE in different gastric cancer cells. **(B)** Protein expression and quantification of RAGE in gastric cancer cells. **(C)** RAGE was stably knocked down in SGC7901 cells. **(D)** The peritoneal nodules in nude mice administered with glucose-derived AGEs for two months. **(E)** The quantification of peritoneal nodules. (^**^P<0.01). **(F)** The inhibitory effect of RAGE knock-down on the AGEs-induced Sp1 and MMP2 expression *in vivo*.

RAGE was reported to mediate the physiological effect of AGEs [12]. We found that the expression of RAGE in cytomembrane of GC cells, such as SGC7901, MNK45, was much higher than that in EGS-1, the normal gastric epithelial cell (Figure 2A, 2B, Supplementary Figure 3). In order to verify the effect of AGEs on GC metastasis, we stably knocked down RAGE expression in SGC7901, with RAGE-specific shRNAs (Figure 2C). And the peritoneal metastasis model of gastric cancer was established with control or RAGE-silenced SGC7901 cells subcutaneously injected into abdomens of nude mice. Compared with RAGE-silenced group, administration of glucose-derived AGEs by peritoneal injection every other day for two months significantly promoted the GC peritoneal spread in control group (Figure 2D, 2E), which verified the *in vivo* positive effect of AGEs on gastric cancer metastasis. And the results of Western blot showed that AGEs treatment can up-regulate the expression of RAGE, Sp1 and MMP2, RAGE knockdown partly reversed AGEs-induced effects (Figure 2F).

### AGEs-RAGE promote gastric cancer cell metastasis *in vitro*

**Figure 3:**
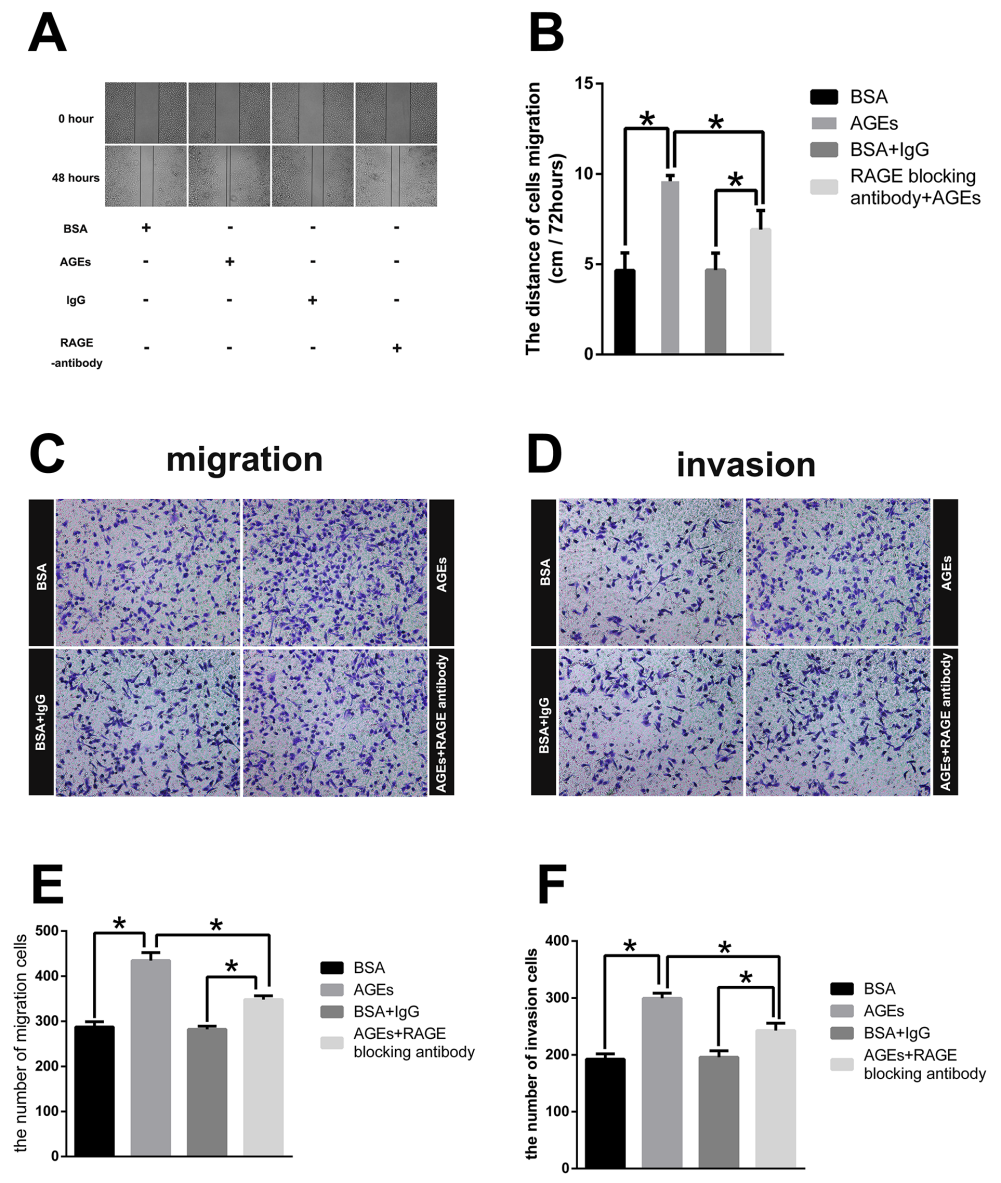
Effect of glucose-derived AGEs on wound healing, invasion, and migration ability in SGC7901 cells (200×) **(A)** Wound healing assays with SGC7901 treated with 100μg/ml AGEs or unmodified BSA was recorded at 0 and 48 hours. **(B)** The mean distances between wound edges of SGC7901 cells at 0 and 48 hours. **(C, D)** Transwell migration and invasion assays for SGC7901 cells treated with 100μg/ml AGEs or unmodified BSA for 2 days. **(E, F)** The number of migration and invasion cells. All experiments were carried out in triplicate. (^**^ *p*<0.05).

In order to confirm the in-vivo effect of AGEs, we treated SGC7901 with 100 μg/ml AGEs for 48 hours. With no effect on cell viability (Supplementary Figure 4), extracellular administration of AGEs enhanced the wound healing, migration, and invasion, which were partially counteracted by the pretreatment of RAGE-specific antibody for 1 hour (Figure 3A–3F). The results of *in vitro* experiments further revealed that AGEs facilitated gastric cancer cell metastasis via binding to RAGE.

### Extracellular treatment of AGEs increases RAGE, Sp1, and MMP2 expression

**Figure 4:**
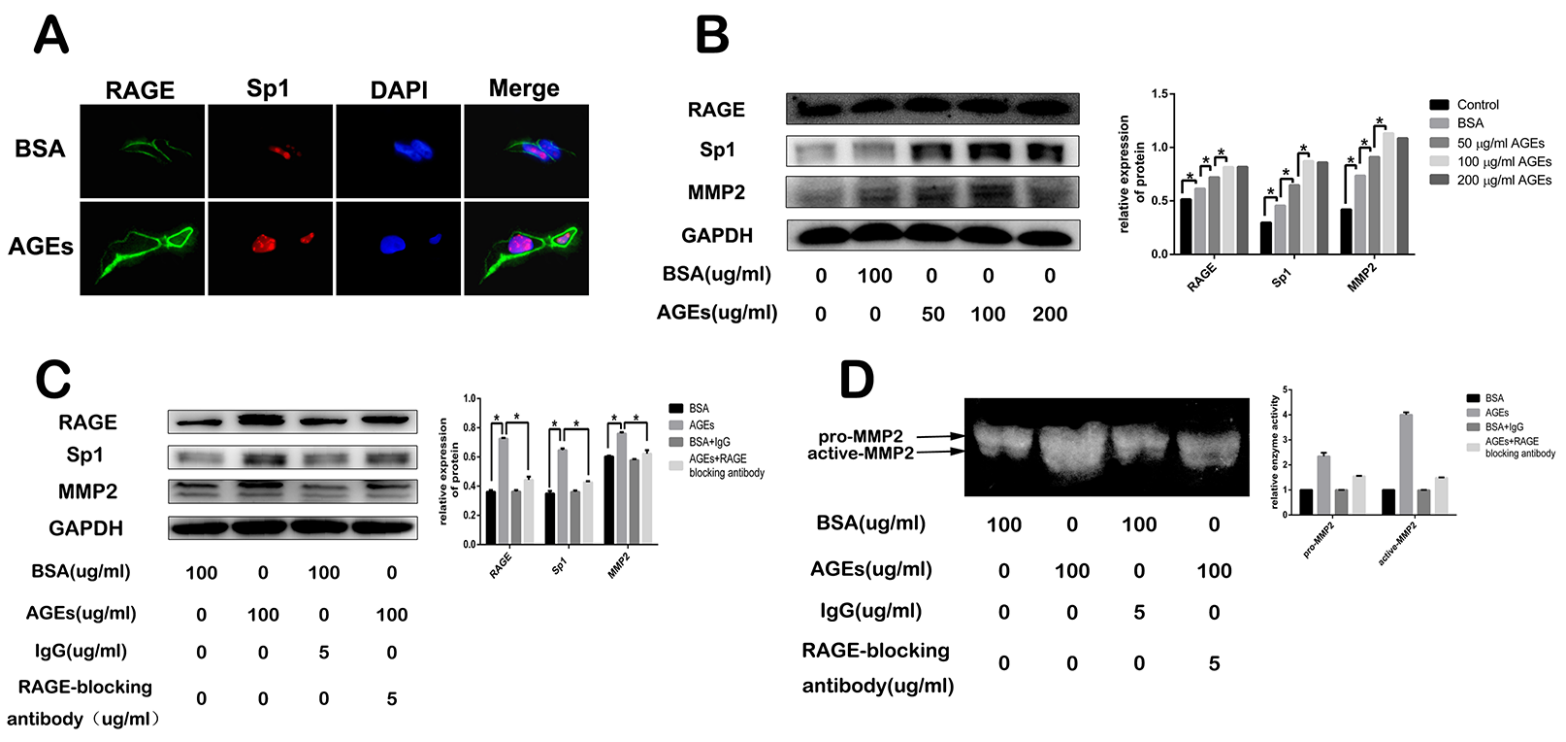
Effect of glucose-derived AGEs on expression of RAGE, Sp1, and MMP2, as well as MMP2 activity in SGC7901 cells **(A, B)** Glucose-derived AGEs upregulated RAGE, Sp1, and MMP2 expression detected by cell immunofluorescence staining and western blot. The scale bar is 25μm. **(C, D)** Inhibitory effect of RAGE blocking antibody on AGEs-induced RAGE, Sp1 and MMP2 expression, as well as MMP2 activity.

We observed that treatment of 100 μg/ml AGEs for 2 days increased RAGE and Sp1 expression in SGC7901 cells (Figure 4A). To further explore the underlying mechanism, different concentrations of AGEs were used to treat the SGC7901 cells for 48 hours. The expression of RAGE, Sp1, and MMP2 increased in a dose-dependent manner (Figure 4B). And AGEs-induced effects reached plateau at the concentration of 100 μg/ml (Figure 4B). However, the pretreatment of RAGE-specific blocking antibody diminished AGEs-induced effects, which suggested that the AGEs-RAGE interaction increased the expression of Sp1 and MMP2 in gastric cancer cells (Figure 4C). The result of gelatine zymography showed that administration of 100 μg/ml AGEs for 48 hours also enhanced matrix metalloproteinases (MMP)-2 activities, which was partially blocked by RAGE neutralizing antibody (Figure 4D).

### Sp1 mediates AGEs-induced gastric cancer metastasis

**Figure 5:**
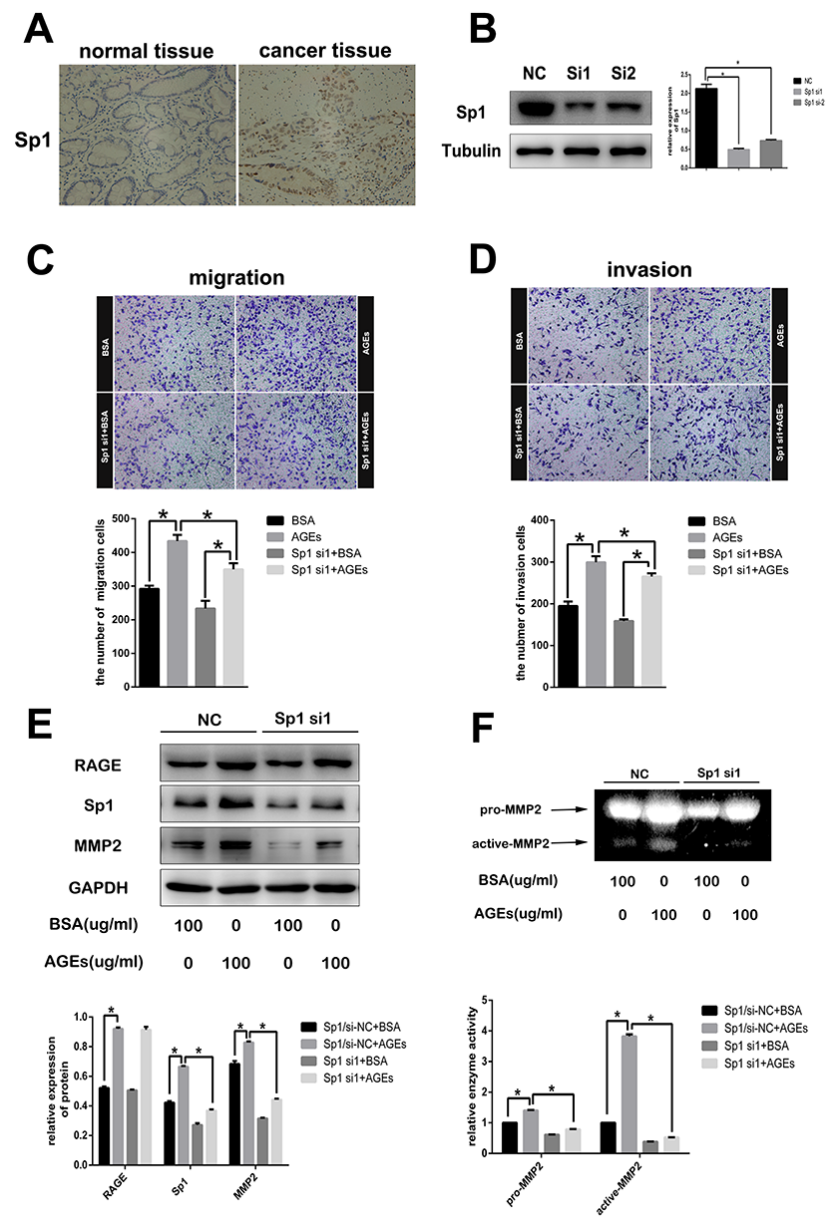
The role of Sp1 on AGEs-induced gastric cancer cells metastasis **(A)** Representative images of immunohistochemistry showed overexpression of Sp1 in human gastric cancer tissues (200×). **(B)** Suppression of Sp1 protein expression in SGC7901 cells with Sp1sepcific siRNAs. **(C, D)** The inhibitory effects of Sp1 knockdown on AGEs-induced cells migration and invasion. **(E, F)** Reduction of AGEs-induced MMP2 protein expression and enzymatic activity in SGC7901 cells by down-regulating Sp1 expression. All operations were repeated three times. (^**^ *p*<0.05).

Sp1 has been shown to be involved in many tumor processes [10]. Here, Sp1 was detected to be over-expressed in gastric cancer tissues (Figure 5A). We reduced Sp1 expression by infecting SGC7901 cells with Sp1-specific siRNAs (Figure 5B). Subsequently, both migration rate and invasion rate of Sp1-silenced SGC7901 cells decreased (Figure 5C, 5D). As a transcriptional factor, Sp1 can regulate many genes expression to promote the progression and poor prognosis of many cancers [10]. As reported, aberrant over-expression of Sp1 in human glioma promoted MMP2-mediated cell invasion [13]. Also, we found that reduction of Sp1 expression decreased the AGEs-induced MMP2 expression (Figure 5E) and MMP2 activity (Figure 5F), without any effect on AGEs-induced RAGE expression, which suggested that Sp1 was a member of AGEs/RAGE/MMP2 cascade.

### AGEs activate ERK to regulate Sp1 expression

**Figure 6:**
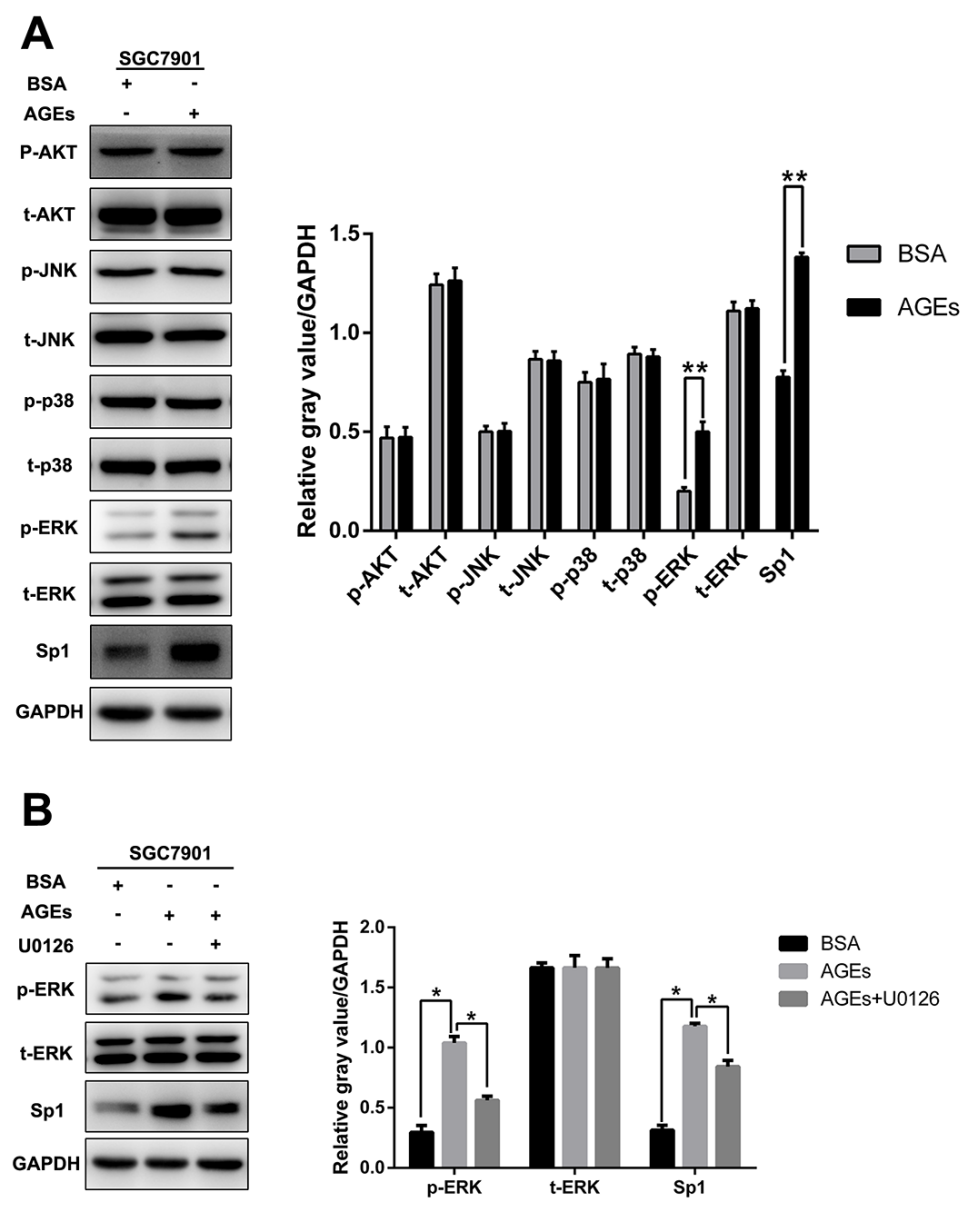
AGEs upregulated Sp1 expression through ERK activation **(A)** The level of p-AKT, p-ERK, p-JUK, p-p38, and Sp1 protein expression. **(B)** Incubation of MEK1/2 inhibitor, U0126 (10μM), for 48 hours reduced AGEs-induced Sp1 expression. (^**^ *p*<0.05).

We observed that, with up-regulating Sp1 protein expression, 100 μg/ml glucose-derived AGEs treatment for 2 days increased the phosphorylation level of ERK in SGC7901 cells with no effect on AKT, JNK and p38(Figure 6A). However, preincubation of MEK1/2 inhibitor, U0126, not only abated the phosphorylation of ERK, but also reduced the AGEs-induced Sp1 expression (Figure 6B). These results suggested that AGEs increased Sp1 expression in SGC7901 cells via augmenting ERK signaling.

## DISCUSSION

Gastric cancer (GC) is the second most common cause of cancer-related death worldwide [14, 15]. How to improve the prognosis of GC is an issue of common concern. The development of gastric cancer is a complex process affected by many factors. Among the predisposing factors are age, smoking, inflammation, Helicobacter pylori, and so on [1]. The identification of other risk factors of gastric cancer and the clarification of potential mechanism can provide an illuminating insight into GC progression. To the best of our knowledge, we firstly found that glucose-derived AGEs were significantly accumulated in cancer tissues and blood of GC patients. AGEs promoted the invasion and migration of GC cells by increasing expression of RAGE, Sp1, and MMP2. The identification of AGEs’ role in progression of gastric cancer and the potential mechanism may help us discover a new risk factor to improve the prognosis of stomach cancer.

As now well known, AGEs abnormally accumulate in diabetic patients with six subtypes, including glucose-derived AGEs (AGE-1), glyceraldehyde-derived AGEs (AGE-2), glycolaldehyde-derived AGEs (AGE-3), methylglyoxal-derived AGEs (AGE-4), glyoxal-derived AGEs (AGE-5), and 3-deoxyglucosone-derived AGEs (AGE-6), which play important roles in diabetic complications [12, 16]. Likewise, AGEs are reported to be involved in many cancer progressions [4, 17]. Via binding to the receptor for advanced glycation end products (RAGE), AGEs strongly promoted proliferation and metastasis of cancer cells, such as primary acute myeloid leukemia (AML) cells, prostate cancer cells, human oral cancer cells, breast cancer cells, and melanoma cells [18-22]. And our research group found that glucose-drive AGEs significantly promoted the growth of colorectal cancer cells by increasing ChREBP expression and nuclear translocation under the glucose-free condition [23]. A lot of factors include hyperglycemia, age, smoking, and inflammation will accelerate the formation and accumulation of advanced glycation end products *in vivo* [2]. Among these factors, age, smoking, and inflammation appear to be “common risk factors” for AGEs accumulation and development of gastric cancer. Here, we found abnormal accumulation of AGEs in cancer tissues and blood of GC patients, which may be caused by “the common risk factors”.

The receptor for advanced glycation end products, popularly known as RAGE, were also indicated to be overexpressed in many cancers including hepatocellular, breast cribriform, colorectal, gastric carcinomas and so on [24]. Moreover, aberrant overexpression of RAGE was positively associated with the invasive and metastatic activity of stomach cancer in Japanese [9]. In the present study, we also found overexpression of RAGE in GC tissues in Chinese Han population, which was likewise significantly associated with lymph node metastasis and TNM stage. And we uncovered that the accumulation of glucose-derived AGEs was one cause of RAGE aberrant overexpression in gastric cancer tissues. AGEs can up-regulate the RAGE expression and activate the multiple RAGE signaling depending on cell type under concern to play the physiological and pathological effects [25]. We found that AGEs treatment increased the expression of Sp1, MMP2, as well as RAGE, in a dose dependent manner. And AGEs-induced effects were reversed by the preincubation with RAGE blocking antibody. RAGE is responsible for the AGEs-induced signal transmission.

Sp1 was detected to be over-expressed in many cancers, such as gastric, breast, pancreatic, glioma, and thyroid cancers, and is closely associated with the stage, invasive potential and metastasis [10]. However, the potential mechanism of Sp1 overexpression in cancer tissues is unclear now. Many factors including HIF1a, ZEB2, and Sp1 itself were reported to be implicated in upregulation of Sp1 protein expression [26-28]. Here, we uncovered that, after binding to RAGE, AGEs upregulated Sp1 expression via activating MEK1/2/ERK pathway, while the treatment of MEK1/2 inhibitor, U0126, reduced the AGEs-induced Sp1 expression in gastric cancer cells by blocking the activation of ERK. Recently, activation of ERK signaling pathway was reported to increase MMP2 expression and promote gastric cancer cells invasion [29]. As a transcriptional factor, Sp1 can regulate many effector molecules including matrix metallopeptidase 2 (MMP2). MMP2 can degrade the type collagen to facilitate the metastasis of cancer cells [30]. AGEs treatment not only increased the MMP2 protein expression, but also enhanced the enzymatic activity of MMP2. Sp1 knockdown reduced the AGEs-induced effects, with no effect on RAGE expression, which suggested that Sp1 mediated AGEs-induced MMP2 expression. As so many cancer-related genes including VEGF, E-cadherin, RECK, and integrin α5 can be regulated by Sp1 [10], there may be other effector molecules implicated in AGEs/RAGE/Sp1 cascade which mediated gastric metastasis. Taken together, glucose-derived AGEs were accumulated in tumor tissues and in the blood of gastric cancer patients without diabetes in Chinese Han population. As a candidate risk factor for gastric cancer, glucose-derived AGEs can promote gastric cancer cells invasion and metastasis via RAGE/ERK/Sp1/MMP2 pathway. Our study may provide a new cue to improve the prognosis of patients with gastric cancer.

## MATERIALS AND METHODS

### Reagents

The primary antibodies for RAGE, MMP2 were obtained from Abcam and antibodies against Sp1, AKT, P-AKT, ERK, p-ERK, JNK, p-JNK, p38, p-p38, Alexa Fluor®488 and Alexa Fluor®594 were purchased from Cell signaling technology. DAPI was purchased from Thermal Fisher Scientific (USA). MEK1/2 inhibitor, U0126, was purchased from Selleckchem (Houston, USA).

### Clinic specimens and measure of serum glucose-derived AGEs

160 sets of gastirc tumors and paired non-tumorous tissues (more than 5 cm away from primary tumor margin) were obtained from patients who underwent curative surgery in Ruijin Hospital affiliated to Shanghai Jiaotong University School of Medicine from 2009 to 2014. The patients didn’t receive any preoperative radiotherapy or chemotherapy before surgery. The pathological stage of tumor was evaluated strictly according to the criteria of UICC TNM classification. Two pathologists independently determined the histological type of tumor in a double–blind way. The blood samples of patients were collected with tubes containing EDTA before surgery. Serum was obtained and preserved at -80°C after blood was centrifuged at 3000 rpm, 4°C, for 5 minutes. The level of serum glucose-derived AGEs was detected with glucose-derived AGEs specific antibody (Abcam, UK) by Western blot. All of the related studies were carried out according to the Code of ethics of the World Medical Association (Declaration of Helsinki) and were approved by Medical Ethics Committee of Shanghai Ruijin Hospital.

### RNA extraction and real-time PCR

RNA extraction of tissue samples and SGC7901 cells and qRT-PCR analysis were carried out as previously described [31]. The forward and reverse primers designed for RAGE were 5′-ACTACCGAGTCCGAGTCTACC-3′ and 5′- CCACCTTATTAGGGACACTGG-3′, respectively. The forward and reverse primers designed for GAPDH were 5′-GAGTCAACGGATTTGGTCGT-3′ and 5′-GACAAGCTTCCCGTTCTCAG-3′, respectively.

### Western blotting analysis

The protein lysates of cells or tissue specimens and Western blotting were performed as previously described [31].

### Immunohistochemistry

Immunohistochemistry was performed as previously described [31].

### Cell culture

The gastric cancer cell line, SGC7901, was kindly provided by Shanghai Digestive Surgery Institute. SGC7901 was cultured in RPMI-1640 with 10% FBS under 37°C, 5% CO2 and saturation humidity. When treated with 100 μg/ml glucose-derived AGEs for 2 days, SGC7901 cells were culture in RPMI-1640 with 2.5% FBS. Glucose-derived AGEs were prepared and purified as previously described [32]. And 5 μg/ml RAGE-blocking antibody (Santa Cruz, USA) or mouse serum lgG (sigma, USA) were added for 1 hour to block the receptor for AGEs, before the treatment of AGEs.

### Cell viability assay

2000 cells were seeded at every well in 96-well plate for 24 hours and were designed with six copies each group. After incubated with serum-poor medium (SPM) supplemented with 2.5% FBS in the presence of 100 μg/ml AGEs or non-modified BSA for different times, cell viability was measured with CCK8 (Beyotime, China). After incubation with CCK8 at 37°C for two hours, OD was assessed at 490 nm using spectrophotometer (Thermo Fisher Scientific, USA).

### Stable gene transfection

In order to stably knock-down RAGE expression of SGC7901 cells, lentiviral expression vectors containing RAGE-specific shRNAs and green fluorescent protein (GFP) were constructed by GeneChem (Shanghai, China). Briefly, SGC7901 cells were seeded on 6-well plates. 24 hours later, cells were transfected with lentiviral expression vectors according to manufacturer’s instructions (Shanghai GeneChem Co., Ltd.). For selection of stable clones, the infected cells were treated with 5 μg/ml puromycin for 7 weeks. The interference efficiency was detected by Western bolt analysis. The base sequences of RAGE shRNA were as follows: S1 forward: 5′-CCGGGTGCCAGGCAATGAACAGGAACTCGAGTTCCTGTTCATTGCCTGGCACTTTTTG-3′, S1 reverse: 5′-AATTCAAAAAGTGCCAGGCAATGAACAGGAACTCGAGTTCCTGTTCATTGCCTGGCAC-3′; S2 forward: 5′-CCGGCACCTGACACATCTTGCAAAACTCGAGTTTTGCAAGATGTGTCAGGTGTTTTTG-3′, S2 reverse: 5′-AATTCAAAAACACCTGACACATCTTGCAAAACTCGAGTTTTGCAAGATGTGTCAGGTG-3′.

### Transwell migration and invasion assay

In order to assess the ability of invasion and migration, transwell migration and invasion assay were carried out as previously described [31].

### Immunofluorescence

2000 cells were plated on every well of Millicell® EZ slides (Millipore, USA) and cultured in complete medium for 24 hours. After treated with or without 100 μg/ml AGEs for 2 days, cells were rinsed with 1×PBS for twice, fixed using 4% paraformaldehyde for 10 minutes at room temperature (30 min for human GC tissues) and then increased permeability of cells using 0.1% Triton X-100 for 5 minutes at room temperature. Incubation of cells or GC tissues with primary antibody overnight at 4°C and subsequently with fluorescent secondary antibody for 1 hour at room temperature were performed in turns. Finally, the slides were washed with 1×PBS for 3 times, subsequently mounted with Prolong Gold with DAPI (Invitrogen, USA) and imaged on fluorescence microscope (Leica, Germany).

### Transient knockdown using siRNAs

Sp1 siRNA were obtained from Obio Technology Co., Ltd (Shanghai, China). The siRNA transfection was carried out using lipofectamine 2000 according to manufacturer’s protocol. The control siRNA sequence was 5′-CCAACAGAUUAUCACAAAUDTDT-3′. The Sp1 siRNA sequence was 5′-AUUUGUGAUAAUCUGUUGGTDTD-3′.

### Gelatin zymography

To assess the matrix metalloproteinase (MMP) activities, the medium used for cell invasion was collected and centrifuged at 1000 rpm, 4°C for 5 minutes. Equivalent volume supernatant was mixed with non-denatured gel sample loading buffer and subjected to electrophoresis on 10% SDS-PAGE gels containing 1mg/ml gelatin (Sigma, USA). The gels were washed with the Novex zymogram renaturing buffer (Invitrogen, USA) for 30 min at room temperature and then incubated in Novex zymogram developing buffer (Invitrogen, USA) at 37°C overnight. The Coomassie brilliant blue staining, photographed and quantified by densitometry with Image J were performed in turns.

### Nude mouse xenograft model

The peritoneal metastasis models of gastric cancer were established in four-week-old male BALB/C nude mice (Institute of Zoology, Chinese Academy of Sciences of Shanghai) as previously described [31]. Three days later, 30 mg/kg sterile AGEs were administrated by intraperitoneal injection every other day for two months. The related operations were approved by the Institutional Animal Care and Use Committee (IACUC) at Shanghai Jiaotong University School of Medicine.

### Statistical analysis

All of experiments were done in triplicate. Data were expressed as mean±SD and analyzed by SAS 8.0 statistical software. Correlations between the level of RAGE expression in GC tissues and clinicopathological parameters were analyzed by chi-square or Fisher’s exact tests. Student’s T test was used to analyze the statistical differences between two groups. *P*<0.05 and *P*<0.01 were respectively considered as statistically significant.

## SUPPLEMENTARY MATERIALS FIGURES



## Abbreviations

AGEs: advanced glycation end products
RAGE: receptor for advanced glycation end products
Sp1: Specificity Protein 1
MMP2: matrix metalloproteinases 2
