# Supplementary material for: Glucose-derived AGEs enhance human gastric cancer metastasis through RAGE/ERK/Sp1/MMP2 cascade
Source: Oncotarget. 2017 Oct 31;8(61):104216–26. doi: 10.18632/oncotarget.22185 (PMC5732800; doi:10.18632/oncotarget.22185)
Supplement: Supplementary file 1 [file oncotarget-08-104216-s001.pdf]

Glucose-derived AGEs enhance human gastric cancer metastasis through RAGE/ERK/Sp1/MMP2 cascade

SUPPLEMENTARY MATERIALS

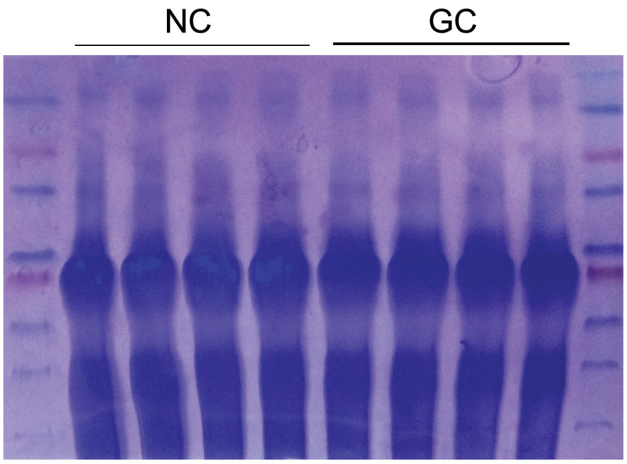

Supplementary Figure 1: Coomassie brilliant blue staining of serum total protein between gastric cancer patients and normal control. The representative figure showed no significant difference in serum total protein between GC (gastric cancer) patients and NC (normal control). All the experiments were repeated in triplicate.

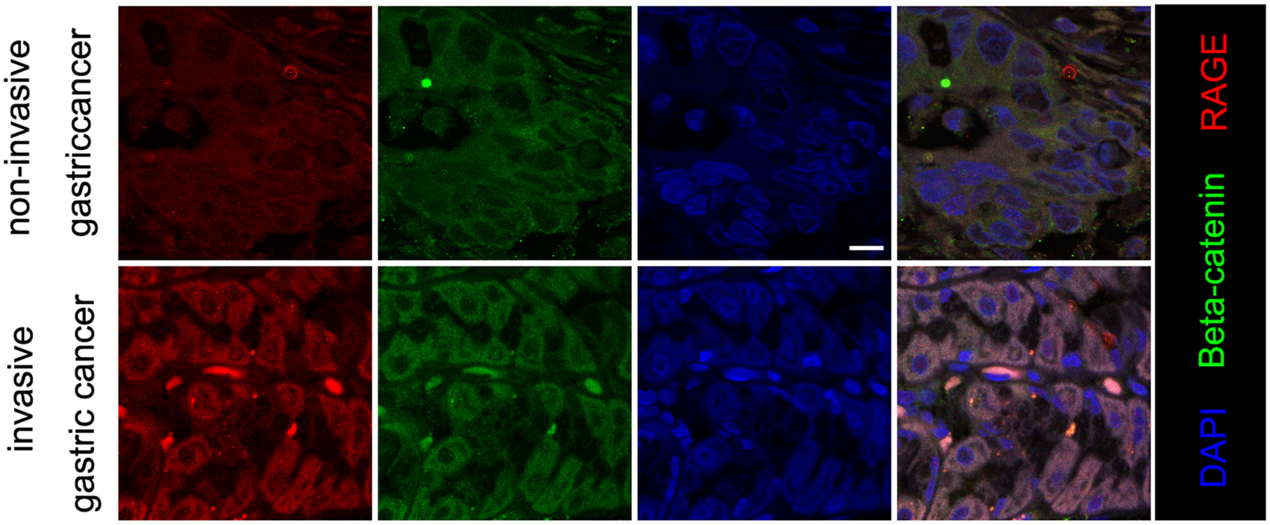

Supplementary Figure 2: RAGE protein expression in non-invasive and invasive gastric cancer tissues detected by immunofluorescence staining and confocal microscopy. The expression level of RAGE in invasive gastric cancer tissues is significantly higher than that of non-invasive gastric cancer tissues. DAPI stains nuclei. Beta-catenin is used as cytomembrane marker. The scale bar is 25 μm.

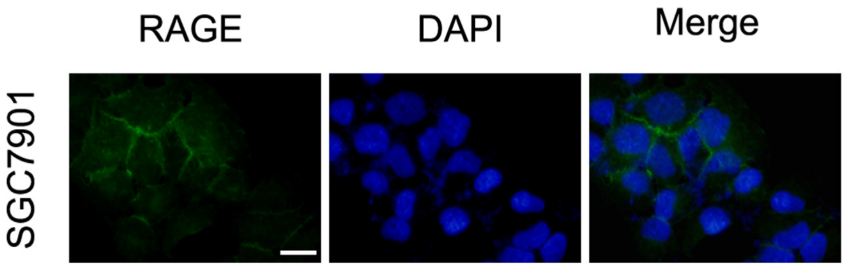

Supplementary Figure 3: RAGE protein expression on the cytomembrane of SGC7901. DAPI stains nuclei. The scale bar is 25 μm.

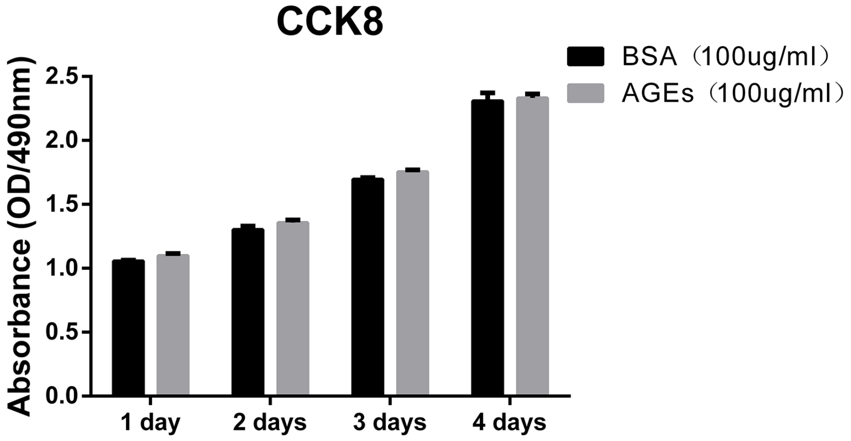

Supplementary Figure 4: Effect of AGEs on cell viability of gastric cancer cells monitored by CCK8. SGC7901 cells are treated with 100 μg/ml glucose-derived AGEs. OD values are determined at 1, 2, 3, and 4 days. * indicates $p<0.05$.
